# Long-Term Consequences of Adolescent Exposure to THC-Rich/CBD-Poor and CBD-Rich/THC-Poor Combinations: A Comparison with Pure THC Treatment in Female Rats

**DOI:** 10.3390/ijms22168899

**Published:** 2021-08-18

**Authors:** Marina Gabaglio, Erica Zamberletti, Cristina Manenti, Daniela Parolaro, Tiziana Rubino

**Affiliations:** 1 Department of Biotechnology and Life Sciences (DBSV) and Neuroscience Center, University of Insubria, 21052 Busto Arsizio, Italy; marina.gabaglio@uninsubria.it (M.G.); cmanenti@uninsubria.it (C.M.); tiziana.rubino@uninsubria.it (T.R.); 2 Zardi-Gori Foundation, Via Curtatone 25, 20122 Milan, Italy; dparolaro@fondazionezardigori.com

**Keywords:** delta-9-tetrahydrocannabinol, cannabidiol, adolescence, females, light cannabis, behavior, GABA, microglia, CB1, GAD67

## Abstract

Cannabis is the most-used recreational drug worldwide, with a high prevalence of use among adolescents. In animal models, long-term adverse effects were reported following chronic adolescent exposure to the main psychotomimetic component of the plant, delta-9-tetrahydrocannabinol (THC). However, these studies investigated the effects of pure THC, without taking into account other cannabinoids present in the cannabis plant. Interestingly, cannabidiol (CBD) content seems to mitigate some of the side effects of THC, at least in adult animals. Thus, in female rats, we evaluated the long-term consequences of a co-administration of THC and CBD at a 3:1 ratio, chosen based on the analysis of recently confiscated illegal cannabis samples in Europe. CBD content is able to mitigate some of the long-term behavioral alterations induced by adolescent THC exposure as well as long-term changes in CB1 receptor and microglia activation in the prefrontal cortex (PFC). We also investigated, for the first time, possible long-term effects of chronic administration of a THC/CBD combination reminiscent of “light cannabis” (CBD:THC in a 33:1 ratio; total THC 0.3%). Repeated administration of this CBD:THC combination has long-term adverse effects on cognition and leads to anhedonia. Concomitantly, it boosts Glutamic Acid Decarboxylase-67 (GAD67) levels in the PFC, suggesting a possible lasting effect on GABAergic neurotransmission.

## 1. Introduction

Cannabis is consumed by more than 182 million people worldwide, with a high prevalence of use among adolescents [1]. It is estimated that in Europe around 15% of people aged 15 to 34 used cannabis in 2019, and the prevalence reaches 19% when only 15- to 24-year-old youths are considered [2].

Adolescence represents a critical period in the refinement of neural circuits [3]. The adolescent brain is highly plastic and undergoes developmental and biological changes that are required for proper behavioral and cognitive development. Because of this still immature state, the adolescent brain can be also more vulnerable to environmental influences, such as cannabis use. Accordingly, exposure to cannabinoids during adolescence has been shown to exert long-term detrimental effects on emotional and cognitive functions in both animal models and humans [4]. Given the high prevalence of cannabis use among adolescents [5] and the fact that cannabis is increasingly viewed as harmless by both adolescents and adults [6], the identification of factors that might influence the adverse impact of cannabis on adolescent brain development and adult behavior is certainly of overwhelming importance. A direct comparison between cannabinoid exposure during adolescence and adulthood has highlighted that an earlier age of initiation of cannabis use represents a major risk factor [4,7]. In addition, the detrimental effects of cannabinoids seem to be dependent on the pattern of use: heavy and frequent cannabis users showing a higher risk for adverse outcomes [4,8]. In addition, in rodents, significant sexual divergence has been reported for both molecular and behavioral outcomes related to cannabis exposure, mainly on domains of function pertaining to the emotional sphere [9]. Another factor that could increase the risk of long-term adverse impact of cannabis on adolescent brain development is related to cannabis potency levels, which seem to depend on the delta-9-tetrahydrocannabinol (THC): cannabidiol (CBD) ratio in cannabis products. THC-rich/CBD-poor cannabis has been associated with increased severity of symptoms, especially in terms of cognitive deterioration and psychosis [10]. In contrast, there is evidence in humans and animals that suggests that the use of CBD-rich/THC-poor strains is associated with fewer adverse effects [11]. However, the investigation of a possible protective effect of CBD towards the adverse effects of THC has focused on the adult population, whereas, to the best of our knowledge, no specific study has addressed this issue in adolescent rodent models using THC:CBD ratios comparable to those present in illegal cannabis.

Based on the observation that CBD would mitigate some of the adverse effects of THC, CBD-rich/THC-poor cannabis varieties have been marketed as non-medicinal products with recreational and self-medication purposes. These preparations have a legally established limit of THC ranging from 0.2 to 1% across countries [12]. Such non-medicinal products contain mainly CBD, at doses lower than those used for medicinal purposes [13]. These so-called “cannabis light” products are perceived as harmless by the general population despite the lack of preclinical evidence in support of their safety, especially after chronic consumption. The high prevalence of cannabis use among adolescents and the availability of these products on the market emphasize the need for research examining the long-lasting effects of chronic CBD-rich/THC-poor cannabis consumption among adolescents on neurobehavioral outcomes.

Based on these premises, in this study, we investigated the possible protective role of CBD when administered in a 1:3 ratio relative to THC, resembling THC-rich/CBD-poor cannabis, during adolescence by comparing the long-term behavioral and neurochemical effects with the ones triggered by pure THC in female rats [14,15,16,17]. Additionally, we evaluated for the first time the long-term effects of chronic administration of a THC/CBD combination reminiscent of CBD-rich/THC-poor cannabis (CBD administered in a 33:1 ratio relative to THC; THC content of 0.3%) in female rats. These ratios were chosen based on the analysis of confiscated illegal cannabis samples in Europe showing a ratio of at least 3 [18] and the average contents of the two cannabinoids in “light cannabis” products [19]. Pure THC, or THC/CBD combinations were administered from postnatal day (PND) 35 to 45 to female Sprague-Dawley rats and, starting from PND 75, cognitive performance and emotional behavior were assessed. In addition to behavioral analysis, neurochemical studies were carried out to investigate the possible effects of CBD and THC co-administration on CB1 receptor levels, gamma-aminobutyric acid (GABA) synthesis as well as on microglia activation state in the prefrontal cortex (PFC), one of the brain regions most affected by adolescent THC exposure [15,16,17,20]. We started our investigation in females because, at least using our protocol of heavy THC exposure during adolescence, we previously observed that female rats developed a different phenotype in the long-term with respect to males. This phenotype was characterized by cognitive deficits and marked alterations in the emotional sphere while male rats developed cognitive deficits and psychotic-like signs without any changes in measures of depression- and anxiety-like behaviors [14].

Findings from this study suggest that the administration of a total THC/total CBD combination with a ratio of 3 can protect against some long-term adverse effects, which are instead triggered by adolescent exposure to pure THC in female rats both at the behavioral and the biochemical levels. In addition, we highlight for the first time that repeated administration of a total THC/total CBD combination with a ratio of 0.33, resembling “light cannabis”, might have long-term adverse effect on the adolescent brain.

## 2. Results

**Figure 1:**
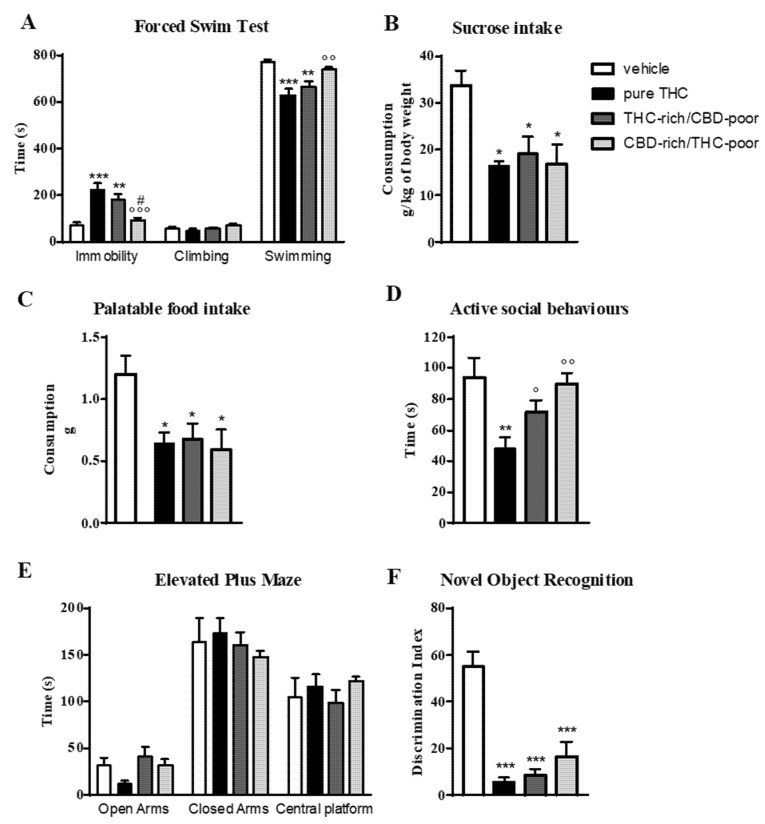
Behavioral responses of adult female rats following adolescent administration of pure THC or THC/CBD combinations in the forced swim test (panel **A**), sucrose intake (panel **B**), palatable food intake (panel **C**), social interaction test (panel **D**), elevated plus maze (panel **E**) and novel object recognition test (panel **F**). Data are expressed as mean ± SEM of 6 animals per group. * *p* < 0.05, ** *p* < 0.01, *** *p* < 0.001 vs. vehicle; ° *p* < 0.05, °° *p* < 0.01, °°° *p* < 0.001 vs. pure THC; # *p* < 0.05 vs. THC-rich/CBD-poor. One-way ANOVA followed by Tukey’s post-hoc test.

Figure 1 shows the effect of adolescent exposure to pure THC, THC-rich/CBD-poor and CBD-rich/THC-poor combinations on emotional behaviors, and short-term recognition memory evaluated in adult female rats.

### 2.1. Emotional Behaviors

In the forced swim test (FST; panel A), as expected, adolescent THC treatment induced a significant increase—of about 214%—in the time spent in immobility with respect to control animals during the test session. This increase was associated with a significant reduction in the time spent in swimming activity. A significant increase in the time spent in immobility compared with control animals (+154%) was also observed after administration of the THC-rich/CBD-poor combination, although this effect was slightly reduced with respect to pure THC. Administration of the CBD-rich/THC-poor combination did not induce any long-term alterations in the FST.

Investigation of sucrose intake (panel B) and palatable food intake (panel C) revealed that, as expected, adolescent THC treatment significantly reduced both sucrose and palatable food intake, by about 49.5 and 48.8%, respectively, compared to vehicle. Administration of a THC-rich/CBD-poor combination induced significant reductions in both sucrose and palatable food intake, and the same effects were also observed after adolescent treatment with a CBD-rich/THC-poor combination.

In the social interaction test (panel D), chronic THC treatment during adolescence impaired sociability, as demonstrated by a significant reduction (−38%) in the time spent in active social behaviors compared to control animals. Sociability deficits were not observed in the long-term after administration of THC-rich/CBD-poor and CBD-rich/THC-poor combinations. A similar effect was also found in the elevated plus maze (EPM) test (panel E). Adolescent THC exposure showed a non-significant trend toward reducing the time spent in the open arms of the maze, with a reduction of about 64.4% with respect to vehicle. A reduction of about 33.3% was also seen in the number of open arms entries with respect to controls (data not shown). Administration of both THC-rich/CBD-poor and CBD-rich/THC-poor combinations did not alter the time spent in the open arms in the long term. No differences were observed in the time spent in closed arms and in the central platform in all experimental groups.

### 2.2. Short-Term Recognition Memory

The time spent exploring the two identical objects during the familiarization phase was not altered in any of the groups analyzed (data not shown). As shown in panel F, adolescent THC administration triggered short-term memory deficits in the novel object recognition (NOR) test, as demonstrated by a significant reduction in the discrimination index of about 89.9% compared to vehicle. Similarly, administration of THC-rich/CBD-poor and CBD-rich/THC-poor combinations induced significant reductions in the discrimination index, of about 84.5% and 70.43%, respectively, compared to controls.

### 2.3. Cannabinoid Type-1 (CB1) Receptor, Glutamic Acid Decarboxylase-67 (GAD67) and Cluster of Differentiation-11b (CD11b) Protein Levels in the Adult Prefrontal Cortex

**Figure 2:**
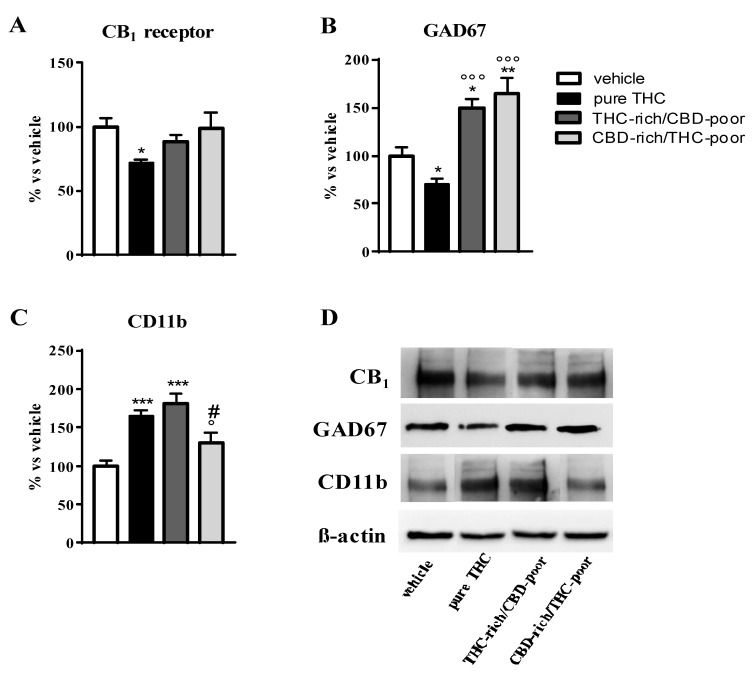
Effect of administration of adolescent THC and THC/CBD combinations on CB1 receptor protein levels (panel **A**), GAD67 protein levels (panel **B**) and CD11b levels (panel **C**) in the adult prefrontal cortex as measured through Western blot analysis. Representative Western blot images are depicted in (panel **D**) and refer to one blot out of six. Data are expressed as mean ± SEM of 6 animals per group. *** *p* < 0.001, ** *p* < 0.01, * *p* < 0.05 vs. vehicle; °°° *p* < 0.001, ° *p* < 0.05 vs. pure THC; # *p* < 0.05 vs. THC-rich/CBD-poor. One-way ANOVA followed by Tukey’s post-hoc test.

Figure 2 shows the effect of adolescent exposure to pure THC, THC-rich/CBD-poor and CBD-rich/THC-poor combinations on CB1 receptor, GAD67 and CD11b protein levels in the adult PFC as evaluated through Western blot analysis.

As expected, chronic THC treatment during adolescence significantly reduced CB1 receptor expression in the PFC—by about 29%—with respect to vehicle-treated rats. No changes in CB1 receptor levels were instead observed after administration of THC-rich/CBD-poor and CBD-rich/THC-poor combinations in the adult PFC (panel A). Adolescent THC treatment significantly reduced GAD67 expression, the main GABA synthetic enzyme, in the PFC, with a reduction of about 30% with respect to vehicle. In contrast, administration of THC-rich/CBD-poor and CBD-rich/THC-poor combinations not only significantly prevented the reduction in GAD67 levels triggered by pure THC, but also significantly enhanced GAD67 expression above control levels, by about 49% and 64%, respectively. Adolescent THC treatment significantly increased the expression of the microglia activation marker, CD11b, in the adult PFC, by about 65% with respect to vehicle. Administration of the THC-rich/CBD-poor combination increased CD11b protein levels by about 81% with respect to controls. In contrast, administration of the CBD-rich/THC-poor combination did not alter CD11b expression in the adult PFC.

### 2.4. Microglia Morphology in the Adult Prefrontal Cortex

To deepen our investigation on the effects of pure THC and THC/CBD combinations on microglia cells, immunohistochemical analysis were carried out using the microglia marker Ionized calcium-binding adapter molecule 1 (Iba1), which stains the whole microglia cell, thus allowing the measurement of morphological parameters related to the activation state of these cells. In fact, microglia under resting conditions show a highly ramified morphology characterized by a small and irregular soma. Upon activation, these cells retract their processes (reducing their cell area and perimeter), and they increase their soma size and roundness [21].

**Figure 3:**
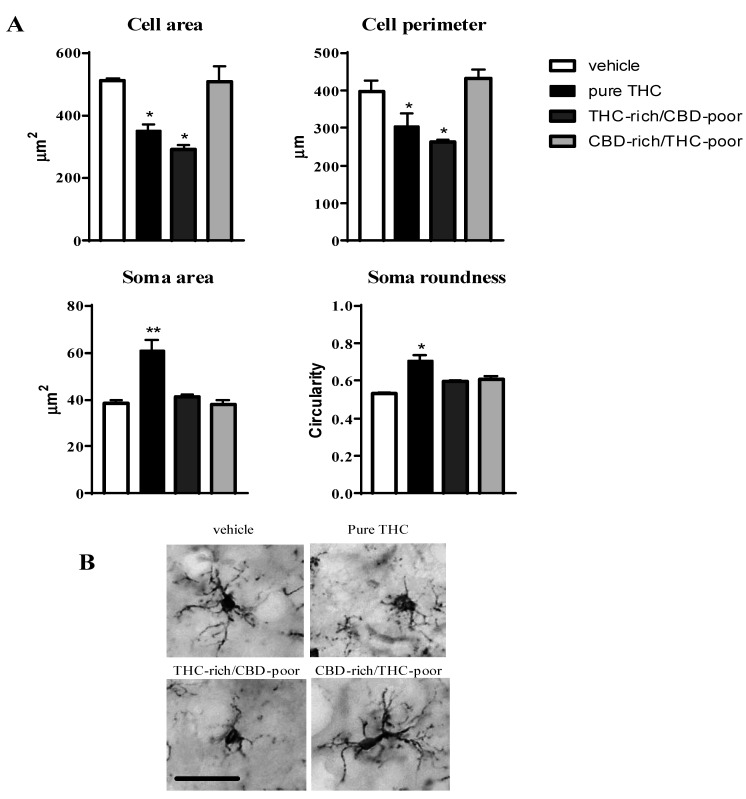
Effect of administration of adolescent THC and THC/CBD combinations on microglia morphology (cell area and perimeter, soma size and roundness (panel **A**) in the adult prefrontal cortex as measured through Iba1 immunostaining. Representative pictures of Iba1-positive cells in the prefrontal cortex are reported in (panel **B**); scale bar = 20 µm. Data are expressed as mean ± SEM of 3 animals per group (36 cells/group). ** *p* < 0.01, * *p* < 0.05 vs. vehicle. One-way ANOVA followed by Tukey’s post-hoc test.

Figure 3 shows the effect of adolescent exposure to pure THC, THC-rich/CBD-poor and CBD-rich/THC-poor combinations on the cell area and perimeter, soma size and roundness of Iba1-positive cells within the adult PFC.

Chronic THC treatment during adolescence significantly reduced the cell area and perimeter of Iba1-positive cells, by about 31.4% and 23.6%, respectively, with respect to vehicle, and increased their soma size (+57.4%) and roundness (+33%). Administration of the THC-rich/CBD-poor combination led to a reduction in the cell area (−42.8%) and perimeter (−33.9%) of microglia cells without affecting their soma morphology, possibly suggesting that a low CBD content in the preparation could partially prevent the THC-induced activation of microglia cells. In contrast, administration of the CBD-rich/THC-poor combination did not induce any changes in microglia morphology in the long term.

### 2.5. Long-Term Behavioral Consequences of Adolescent CBD (5 mg/kg; Twice a Day; PND 35–45) Treatment in Female Rats

To assess whether the long-term behavioral effects triggered by chronic CBD-rich/THC-poor combination administration in adolescent female rats were attributable to CBD or THC content in the preparation, we replicated pharmacological treatments and behavioral testing in a separate cohort of rats by also investigating the effects of chronic CBD administration (5 mg/kg; twice a day; PND 35–45).

**Figure 4:**
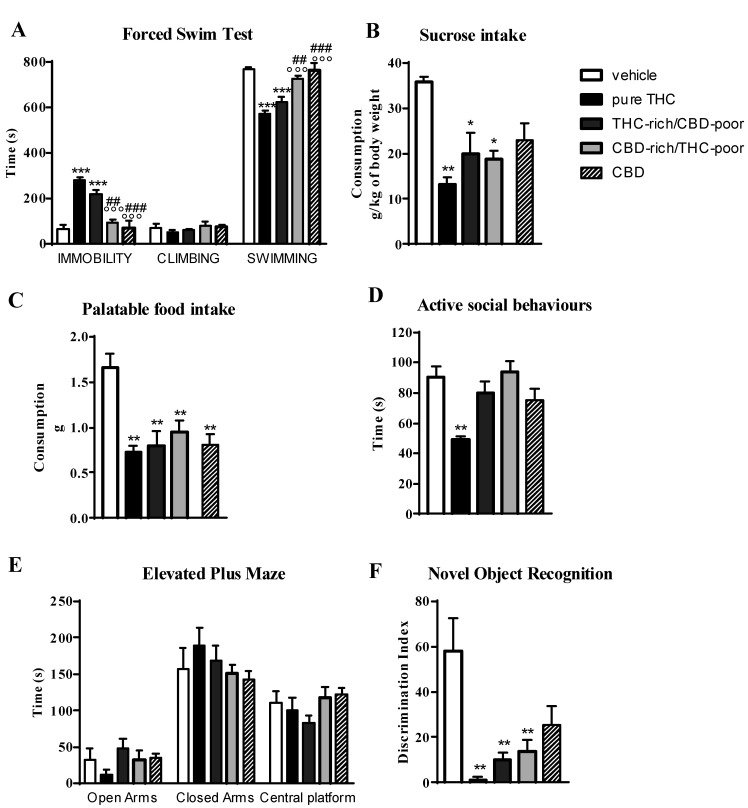
Behavioral responses of adult female rats following adolescent administration of pure THC, THC/CBD combinations or CBD in the forced swim test (panel **A**), sucrose intake (panel **B**), palatable food intake (panel **C**), social interaction test (panel **D**), elevated plus maze (panel **E**), novel object recognition test (panel **F**). Data are expressed as mean ± SEM of 4 animals per group. * *p* < 0.05, ** *p* < 0.01, *** *p* < 0.001 vs. vehicle; °°° *p* < 0.001 vs. pure THC; ### *p* < 0.001, ## *p* < 0.01 vs. THC-rich/CBD-poor. One-way ANOVA followed by Tukey’s post-hoc test.

Figure 4 shows the effect of adolescent exposure to pure THC, THC-rich/CBD-poor and CBD-rich/THC-poor combinations, or CBD alone, on emotional behaviors and short-term recognition memory evaluated in adult female rats.

In the FST (panel A), adolescent THC treatment and the administration of the THC-rich/CBD-poor combination significantly increased the time spent in immobility, by about 330% and 235.7%, compared to control animals, and simultaneously reduced the time spent in swimming activity. Administration of the CBD-rich/THC-poor combination or CBD alone did not induce any long-term alterations in the FST.

As reported in Panel B, adolescent THC treatment, administration of the THC-rich/CBD-poor combination and administration of the CBD-rich/THC-poor combination significantly reduced sucrose intake, by about 63%, 44% and 48%, respectively, compared to vehicle. A reduction in sucrose intake of about 36%, although not significant, was observed after chronic administration of CBD in female rats. Similar effects were observed on palatable food intake (Panel C), where significant reductions were reported in all experimental groups.

In the social interaction test (panel D), chronic THC treatment during adolescence significantly reduced—by about 45%—the time spent in active social behaviors compared to control animals. Sociability deficits were not observed in the long-term after administration of THC-rich/CBD-poor and CBD-rich/THC-poor combinations as well as after CBD administration. A similar effect was also found in the EPM test (panel E). Adolescent THC exposure showed a non-significant trend toward, reducing the time spent in the open arms of the maze—by about 65.3%—with respect to vehicle. Administration of both THC-rich/CBD-poor and CBD-rich/THC-poor combinations and treatment with CBD alone did not alter the time spent in the open arms in the long term. No differences were observed in the time spent in closed arms and in the central platform in all experimental groups.

In the NOR test (Panel F), adolescent THC treatment, administration of the THC-rich/CBD-poor combination and administration of the CBD-rich/THC-poor combination significantly reduced short-term recognition memory, by about 97.7%, 82.7% and 76.7%, respectively, compared to vehicle. A reduction in the discrimination index of about 56%, although not significant, was observed after chronic administration of CBD in female rats.

## 3. Discussion

These results indicate the presence of lasting detrimental effects of a chronic treatment with pure THC during adolescence on adult behavior of female rats, thus confirming our previous findings [14,16,22]. In particular, adult female rats chronically exposed to high doses of THC during adolescence display depression- and anxiety-like behaviors, including increased immobility in the FST, anhedonia, reduced social behavior, as well as short-term memory deficits in the NOR test. This is also in line with multiple studies from both humans and animals suggesting that exposure to THC during adolescence has the potential to produce lasting alterations in neurobiology and behavior whose magnitude seems to depend on dosing and timing of THC administration [23]. Notably, research evidence suggests a dose–response relationship for the association between adolescent THC exposure and detrimental effects on brain and behavior, as exposure to THC during adolescence at doses suggestive of a low to moderate dose exposure in humans resulted only in mild or even no long-term deficits in affective or cognitive behavior [24,25].

One of the main criticisms of these studies is that they investigated the effects of pure THC without taking into account the compresence of other cannabinoids in the cannabis plant, which might modulate the overall outcome. In particular, it has been suggested that CBD content in the plant could mitigate some of the side effects of THC, at least in adults [10,26].

In this study, we evaluated the long-term consequences of a concomitant administration of THC and CBD at a ratio of 3, which was chosen based on the analysis of recently confiscated illegal cannabis samples in Europe [18]. The results obtained indicate that the long-term detrimental effects of pure THC were partially prevented when a THC-rich/CBD-poor combination was administered to adolescent female rats. In particular, a protective action of CBD was observed on measures of anxiety-like behaviors. However, depressive-like behavior in the FST, anhedonia and short-term memory deficits in adult female rats were also present following administration of this THC-rich/CBD-poor combination during adolescence. These data suggest that CBD could mitigate some of the long-term adverse effects of THC when exposure occurs in female rats during adolescence, and the protective effect seems to be particularly relevant when anxiety behaviors are considered. We cannot exclude that a lower THC/CBD ratio could result in a more marked protection. Accordingly, when a THC/CBD ratio of 1 was used, in addition to anxiety behaviors, THC-induced cognitive deficits were also prevented, at least in adolescent male mice [27].

In several European countries, a new market for products containing mainly CBD, with very low THC levels (called “light cannabis” or “CBD-rich cannabis”), is emerging. CBD products can be legally distributed if the THC concentration remains below a specified level, ranging from 0.2 to 1% across countries [12]. As there are no regulations on CBD concentration, products with remarkably different CBD concentrations are legally available.

An emerging “light cannabis” market needs to be considered with caution from a public health perspective. As a legal alternative to cannabis with higher THC levels, depending on the users’ appreciation of the psychoactive properties of “light cannabis” products that do not cause the usual “high”, it is possible that a number of young cannabis users may shift to these products. In addition, some teens may initiate use of “light cannabis” products for either recreational or self-medication purposes. In this work, we investigated, for the first time, possible long-term consequences of adolescent administration of a CBD-rich/THC-poor combination at a ratio of 0.33 and with a total THC content of 0.3%, resembling the average contents found in “light cannabis” products available in Italy [19]. Chronic treatment with this CBD-rich/THC-poor combination during adolescence in female rats did not induce depressive-like behaviors in the FST, social behavior deficits nor anxiety-like behaviors in the EPM at adulthood. In contrast, we observed the development of short-term memory deficits and anhedonia in the long term. These data provide scientific evidence for possible long-term adverse consequences of chronic exposure to CBD-rich/THC-poor combinations during adolescence. Although not psychotomimetic, CBD is clearly a psychoactive compound and we speculate that some of its actions might also have detrimental effects on adolescent brain maturation, possibly by interfering with normal developmental trajectories. Accordingly, the same behavioral alterations (i.e., short-term memory deficits and anhedonia) were observed at adulthood after chronic CBD treatment in adolescent female rats.

At the end of behavioral tests, we tried to identify possible neurochemical correlates of the long-term effects triggered by CBD and THC combinations in female rats in comparison with pure THC treatment. We started our investigations based on some of the most consistent long-term changes reported after adolescent THC exposure and associated with the development of THC-induced behavioral alterations: the downregulation of CB1 receptors [15,28], the presence of microglia activation [17], and the alteration of GABA synthesis [16,29]. We focused our analysis on the PFC as previous studies identified this brain region as one of most affected by adolescent THC exposure [15,20]. In addition, brain development continues throughout adolescence, especially in cortical regions, and involves dramatic changes in gross morphology with a loss of gray and a rise in white matter [30,31]. The PFC is known to reach full development late in adolescence, and alterations in its maturation have been implicated in several psychiatric disorders, such as schizophrenia and depression [32]. Indeed, endophenotypes for both these disorders were described in adult female rats exposed to THC during adolescence [14].

Adolescent exposure to THC downregulated CB1 receptor levels in the adult PFC, in line with previous findings [15]. The long-term downregulation of CB1 receptors was prevented when a THC-rich/CBD-poor combination was administered, suggesting a protective effect of CBD also at the biochemical level. No long-term effects on CB1 receptor expression were observed after administration of the CBD-rich/THC-pure combinations.

Activation of microglia within the adult PFC has also been reported as a consequence of adolescent THC exposure [17] and was confirmed by the present results, as demonstrated by the increased expression of the activation marker, CD11b, and changes in Iba1-posive cell morphology, including reduced cell area and perimeter as well as increased soma size and roundness. The administration of the THC-rich/CBD-poor combination increased the expression of the activation CD11b, suggesting an activation of microglia cells. However, the investigation of microglia morphology in these animals provided further information on the activation state of these cells, suggesting that the activation of microglia could be slightly reduced with respect to the one triggered by pure THC. In fact, only alterations in the cell area and perimeter of Iba1-positive cells were present without any changes in soma size and roundness, possibly suggesting an intermediate phenotype of these cells, and thus raising the possibility that the low CBD content present in the preparation could have partially prevented microglia activation. Finally, administration of the CBD-rich/THC-poor combination did not affect microglia activation in adulthood, neither in terms of CD11b expression nor microglia morphology.

In the adult PFC, pure THC treatment during adolescence led to a reduction in the main enzyme responsible for GABA synthesis, GAD67, which was in line with previous findings [16,29] and suggestive of a reduced inhibitory tone in the long term. In contrast, administration of both THC/CBD combinations boosted GAD67 levels above control levels in the same brain region, possibly suggesting a potentiation of GABAergic signaling in the adult PFC. The functioning of the PFC relies on a balance between excitatory and inhibitory neurotransmission. This excitatory/inhibitory (E/I) balance arises from a complex interplay of heterogeneous glutamatergic and GABAergic neurons interacting within a local circuit [32]. In particular, GABAergic interneurons that express the calcium-binding protein parvalbumin (PV) are major regulators of E/I balance and play an important role in prefrontal-dependent emotional and cognitive behaviors [33]. Dysregulations in prefrontal E/I balance were frequently implicated in neuropsychiatric conditions such as schizophrenia, as supported by postmortem analyses that revealed abnormalities in the expression of PV and GAD67 [34]. PFC-related dysfunctions in GABAergic signaling represent a central pathological feature of schizophrenia and seem to be crucially involved in the development of neuronal and behavioral disturbances induced by chronic THC exposure during adolescence [35]. Based on this evidence, it is likely that alterations in GABA synthesis triggered by the administration of different CBD/THC combinations in adolescence could lead to E/I imbalances within the PFC, which might contribute to the cognitive abnormalities observed in these animals. However, a functional evaluation of excitatory and inhibitory transmission is clearly needed to address this hypothesis.

## 4. Materials and Methods

### 4.1. Animals

Female Sprague Dawley rats, aged 28 days at the time of arrival, were obtained from Charles River laboratories S.R.L. (Calco, Lecco, Italy) and were housed in clear plastic cages on a 12 h light-dark cycle (lights on 08:00 h), at a temperature of 22 ± 2 °C and in a humidity-controlled environment (50 ± 10%), with a plastic tube for environmental enrichment. All animals had free access to food (standard laboratory pellets) and water. All experiments took place during the light phase between 09:00 and 15:00. Experimental procedures were performed in accordance with the guidelines released by the Italian Ministry of Health (D.L. 2014/26), and the European Community directives regulating animal research (2010/63/EU). The research project and research procedures were examined and approved by the Body for the Protection of Animals (OPBA) at the University of Insubria, and protocols were then approved by the Italian Minister for Scientific Research. All efforts were made to minimize the number of animals used and their suffering.

### 4.2. Pharmacological Treatments

**Figure 5:**
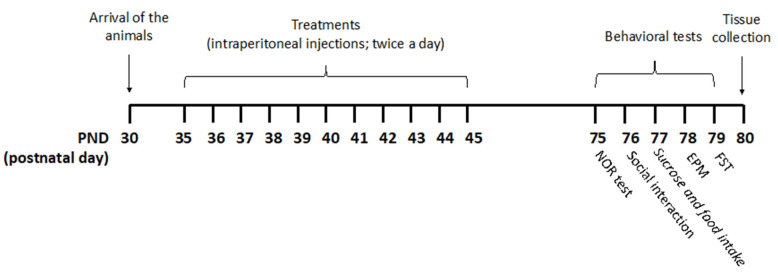
Treatment schedule. Rats received pure THC, THC/CBD combinations, or vehicle twice a day via intraperitoneal (i.p.) injections during the adolescent period from postnatal day (PND) 35 to PND 45. THC/CBD ratios of 3 and 0.33, reminiscent of THC-rich/CBD-poor and CBD-rich/THC-poor cannabis, respectively, were used. For the CBD-rich/THC-poor combination, a THC content of 0.3% was considered. Animals were divided into four experimental groups: vehicle (ethanol: Kolliphor: saline 1:1:18); pure THC (2.5 mg/kg—PND 35–37; 5 mg/kg—PND 38–41; 10 mg/kg—PND 42–45); THC-rich/CBD-poor (THC 2.5 mg/kg + CBD 0.83 mg/kg—PND 35–37; THC 5 mg/kg + CBD 1.66 mg/kg—PND 38–41; THC 10 mg/kg + CBD 3.32 mg/kg—PND 42–45); CBD-rich/THC-poor (THC 0.15 mg/kg + CBD 5 mg/kg). Starting from PND 75, rats underwent behavioral assessment on five consecutive days. Twenty-four hours after the last test, brain tissues were collected for subsequent biochemical analysis.

The experiments were carried out according to the timeline reported in Figure 5.

Rats received pure THC, THC/CBD combinations, or vehicle twice a day via intraperitoneal (i.p.) injections during the adolescent period from postnatal day (PND) 35 to PND 45. Drugs were freshly prepared daily and dissolved in ethanol, kolliphor EL and saline (1:1:18). A volume of injection of 5 mL/kg was used.

According to the transformation of human-equivalent doses proposed by the Food and Drug Administration (FDA), and considering an average THC content of herbal cannabis of about 10.22% in Europe [36], the lowest dose (2.5 mg/kg) corresponded to half of a joint, the second one (5 mg/kg) to one joint, and the highest one (10 mg/kg) to two joints. THC/CBD ratios of 3 and 0.33, reminiscent of THC-rich/CBD-poor and CBD-rich/THC-poor cannabis, respectively, were chosen based on the analysis of 531 confiscated illegal cannabis samples in Europe [18] and the average THC/CBD ratio in cannabis-light products [19]. For CBD-rich/THC-poor combination, a THC content of 0.3% was considered. Each single dose refers to a single administration and animals were treated twice a day.

Rats were divided into four experimental groups as follows:

- Vehicle (ethanol: Kolliphor: saline 1:1:18);
- pure THC (2.5 mg/kg—PND 35–37; 5 mg/kg—PND 38–41; 10 mg/kg—PND 42–45);
- THC-rich/CBD-poor (THC 2.5 mg/kg + CBD 0.83 mg/kg—PND 35–37; THC 5 mg/kg + CBD 1.66 mg/kg—PND 38–41; THC 10 mg/kg + CBD 3.32 mg/kg—PND 42–45);
- CBD-rich/THC-poor (THC 0.15 mg/kg + CBD 5 mg/kg).

### 4.3. Behavioral Tests

#### 4.3.1. Novel Object Recognition (NOR) Test

The experimental apparatus used for the NOR test was an open-field box (43 × 43 × 32 cm) made of Plexiglas, placed in a dimly illuminated room. The experiment was performed and analyzed as previously described [16]. Briefly, each animal was placed in the arena and allowed to explore two identical previously unseen objects for 5 min (familiarization phase). After an interval of 3 min, one of the two familiar objects was replaced by a novel, previously unseen object and rats were returned to the arena for the 5-min test phase. The arena was cleaned between animals with 0.1% acetic acid. During the test phase, the time spent exploring the familiar object (Ef) and the new object (En) was video-taped and recorded separately by two observers who were blind to the treatment groups, and the discrimination index was calculated as follows: [(En − Ef)/(En + Ef)] × 100.

#### 4.3.2. Social Interaction Test

The test was carried out as previously reported [16]. On the day of testing, each animal was habituated for 10 min in the test arena, an open-field box (60 × 60 × 60 cm) made of Plexiglas. During the test session, each animal was allowed to freely explore an unfamiliar congener in the arena for 10 min. The arena was cleaned with 0.1% acetic acid and dried after each trial. Active social behaviors were defined as sniffing, following, grooming, mounting, and nosing. Aggressive behaviors were defined as attacking, biting, tail rattling, and aggressive grooming. The whole testing phase was videotaped, analyzed by two observers who were blind to the treatment groups, and the time spent in social behaviors and the number of aggressive behaviors were recorded.

#### 4.3.3. Forced Swim Test (FST)

Animals were tested in a modified version of the FST with only the first session of swimming, as previously reported [16], to measure a pre-existing behavioral deficit induced by the pharmacological manipulation. Briefly, rats were forced to swim for 15 min inside a clear 50 cm tall, 20 cm diameter glass cylinder filled to 30 cm with 25 °C water. The session was videotaped for later analysis of the following parameters: immobility (time spent by the animal floating in the water making only those movements that were necessary to keep the head above water), swimming (active swimming movements to the center of the cylinder), climbing (forceful thrashing movements with forelimbs against the walls of the cylinder). The time spent in each of these behaviors was measured by an experimenter who was blind to the treatment groups.

#### 4.3.4. Elevated Plus Maze (EPM)

The elevated plus-maze apparatus consisted of two opposite open arms (50 × 10 cm) and two closed arms (50 × 10 × 40 cm) that extended from a common central platform (10 × 10 cm). The apparatus was elevated to a height of 50 cm above floor level and placed in the center of a quiet room under dim light. Rats were placed individually onto the center of the apparatus, and the time spent on each arm and entries onto each arm (arm entry = all four paws into an arm) were measured for 5 min.

#### 4.3.5. Sucrose and Palatable Food Intake

On the day of testing, animals were food and water deprived for 4 h in their home cage. Afterward, rats were housed individually in a cage presenting a drinking bottle containing 100 mL 1% sucrose solution for 2 h. Sucrose solution intake was measured by weighting the bottles before and after the test, and total intake was expressed as grams/kilograms of body weight over the 2 h period. For palatable food intake, animals were housed individually and a petri dishes filled up with palatable food (a snack food made of corn flour, hydrogenated vegetable fat and cheese powder) was presented to the animals during a 5-min test session. Food intake was measured and expressed as total grams consumed over the 5 min period.

### 4.4. Biochemical Studies

Twenty-four hours after the last behavioral test, all the animals were euthanized, their brain tissues were collected, and they were randomly assigned to different procedures for subsequent biochemical analysis. All females received vaginal smears for estrous cycle monitoring: 79% of them were in the diestrus phase while the remaining 21% were in the estrus phase at the time of tissue collection. For western blot analysis, the PFC was dissected, frozen in liquid nitrogen and stored at −80 °C. For immunohistochemistry, the brains were quickly removed, post-fixed in 4% paraformaldehyde (PFA) in 100 mM phosphate buffer (PBS), at pH 7.4, for 48 h, and cryoprotected in 30% sucrose for a minimum of 24 h. Coronal sections were serially collected using a Leica cryostat CM1510 set to 40 µm thickness and a −20 °C chamber temperature.

### 4.5. SDS-Page and Western Blot Analyses

For protein lysate, the PFC was homogenized in an appropriate volume of ice-cold buffer (10 mM Hepes pH 7.5, 1.5 mM MgCl_2_, 10 mM KCl, 2 mM DTT, 1 mM PMSF, 1 mM EDTA, 1 mM EGTA, 2 mM sodium orthovanadate, 50 mM NaF, 10 mM sodium pyrophosphate, 0.5% Triton and protease inhibition cocktail) and centrifuged at 13,000 rpm at 4 °C for 3 min. The supernatant was used as total protein lysate and protein concentrations were determined according to the Micro-BCA assay kit (Pierce, Rockford, IL, USA). Protein lysates were prepared in boiling sodium dodecyl sulfate (SDS) sample buffer and equal amounts (30 µg) of total protein were run on a 10% SDS-polyacrylamide gel. The proteins were then transferred to polyvinylidene difluoride (PVDF) membranes, blocked for 2 h at room temperature in 5% dry skimmed milk in Tris-buffered saline (TBS)-tween 20 (0.1%) before incubation overnight at 4 °C with the primary antibody. The following primary antibodies were used: rabbit polyclonal anti-CB1 (1:1000; CaymanChemical, AnnArbor, MI, USA) and rabbit polyclonal anti-CD11b (1:1000; Novus Biologicals, Littleton, CO, USA), mouse monoclonal anti-GAD67 (1:1000; Millipore, Billerica, MA, USA). Bound antibodies were detected with horseradish peroxidase (HRP) conjugated secondary antibody for 1h at room temperature and visualized using WESTAR ECL Substrate for Western Blotting (Cyanagen, Bologna, Italy), and bands were detected with a G-Box XT camera (Syngene, Cambridge, UK). For detection of β-actin, the blots were stripped with Renew Stripping Buffer (Cyanagen, Italy) and re-blotted with mouse monoclonal anti-β-actin (1:2000; Sigma Aldrich, Saint Louis, MO, USA) overnight at 4 °C and visualized as described above. For densitometry, the optical density of the bands was quantified using ImageJ software (NIH, Bethesda, MD, USA) and normalized to controls. Six different blots were run, and all experimental groups were loaded on each of them. All experiments were performed in duplicate. Given differences in exposure times during the acquisition at the G-Box tool, data were expressed as % of vehicle to allow comparison between blots. This was calculated by setting the value corresponding to the mean arbitrary units of all vehicles at 100, and the arbitrary units for each lane of the blots were converted accordingly.

### 4.6. Immunohistochemistry

Free floating sections, which contained the PFC, were washed three times in 0.05% Tryton X-100 in PBS, incubated with 3% normal goat serum in PBS for 45 min at room temperature, and then incubated overnight at 4 °C with rabbit anti-IBA1 antibody (1:1000, Wako, Neuss, Germany) diluted in blocking solution. No further controls for specificity were performed in addition to the ones provided by the manufacturer. After blocking peroxidase activity with 0.3% H_2_O_2_ in PBS for 15 min, sections were washed and incubated for 4 h at room temperature with HRP-conjugated goat anti-rabbit antibody (1:500, Immunological Sciences, Roma, Italy). The peroxidase activity was revealed with 0.05% diaminobenzidine (DAB) and 0.03% H_2_O_2_ in TBS for 10 min. After several washes in PBS, sections were mounted on gelatine-coated slides, dehydrated and cover slipped. For each animal, a complete series of one-in-six sections (240 mm apart) through the PFC was analysed. Digital Images were captured using Retiga R1 CCD camera (QImaging, Surrey, BC, Canada) attached to an Olympus BX51 (Tokyo, Japan) polarizing/light microscope. Ocular imaging software (QImaging, Surrey, BC, Canada) was used to import images from the camera. Images of microglia cells in the PFC were acquired by first delineating the brain sections and the region of interest at low magnification (×4 objective) and the outlines of the region of interest were further refined under a ×40 objective. Three rats per experimental group (four sections/rat; 12 cells/rat; 36 cells/group) were analyzed. The morphometric analysis was carried out in DAB-stained microglial cells labelled with IBA1 antibody. For this purpose, cells were selected and cropped according to the following criteria: (1) random selection in the prefrontal cortex; (2) no overlapping with neighboring cells; (3) complete soma and branches (at least apparently). Selection was conducted in a manner that was blinded to the treatment. A total of twelve cells were analyzed from each animal. Each grayscale single cell cropped image was processed in a systematic way to obtain a binary image, using the same threshold for all pictures. The binary image was edited to clear the background and transformed into a filled shape and its pairwise outline shape, which were used for measurements of morphological parameters. Analysis was performed using FIJI free software (NIH, Bethesda, MD, USA). Four parameters, measured on the filled and outlined processed images obtained as described previously [21], were analyzed: cell area, cell perimeter, roundness of the soma and soma area.

### 4.7. Statistical Analysis

Data analysis was performed using Prism 5.0 software (Graph-Pad Software, San Diego, CA, USA). Behavioral and biochemical results were expressed as mean ± SEM and were analyzed by one-way ANOVA, followed by Tukey’s post-hoc test. The level of statistical significance was set at *p* < 0.05.

## 5. Conclusions

This study demonstrates that CBD content is able to modulate some of the long-term behavioral alterations induced by adolescent exposure to THC in female rats, especially when anxiety-like behaviors are considered. A protective effect is also observed at the biochemical level, where CBD seems to mitigate long-term changes in CB1 receptor and microglia activation triggered by THC in the PFC. In addition, our findings highlight for the first time that repeated administration of CBD-rich/THC-poor combinations, at a ratio resembling “light cannabis”, might also have long-term adverse effects on the adolescent brain. In the adult PFC, the administration of CBD-rich/THC-poor combinations boosted GAD67 levels, suggesting a possible lasting effect on GABAergic transmission within this brain region. To date, there is a paucity of research on the manner by which CBD may affect the actions of THC across a wide range of behavioral and biochemical effects. CBD is clearly a molecule with multi-target effects whose pharmacological actions might arise from many different mechanisms. The amount of CBD administered, the ratio of CBD to THC and the timing of administration seem to be important in determining the possible effects of CBD.

One major limitation of this study is the lack of information on possible effects of CBD in males. Given the high prevalence of cannabis use among male teens, the investigation of long-term effects of CBD on THC-induced behavioral and neurobiological alterations in males is certainly of overwhelming importance and must represent a major goal of future investigations. Moreover, studies in males might highlight a different sensitivity of the adolescent brain to CBD with respect to females, paving the way for additional investigations on the possible molecular underpinnings.

This study is descriptive in nature and took into account only limited biochemical targets. Additional research should clearly establish potential mechanisms of action, and further investigate possible harms related to the chronic consumption of CBD-rich/THC-poor products among the adolescent population, in order to avoid possible negative public health effects and to introduce regulations for this emerging market that are based on scientific evidence.
